# From complexity to commercial readiness: industry insights on bridging gaps in human-robot interaction and social robot navigation

**DOI:** 10.3389/frobt.2025.1711675

**Published:** 2025-12-16

**Authors:** Lina Moe, Benjamin Greenberg

**Affiliations:** 1 Bloustein School of Planning and Public Policy, Rutgers University, New Brunswick, NJ, United States; 2 Department of Mechanical Engineering, Rutgers University, Piscataway, NJ, United States

**Keywords:** mobile robots, navigation, industry, interview, qualitative research, person detection

## Abstract

This paper examines the evolving landscape of mobile robotics, focusing on challenges faced by roboticists working in industry when integrating robots into human-populated environments. Through interviews with sixteen industry professionals specializing in social mobile robotics, we examined two primary research questions: (1) What approaches to person detection and representation are used in industry? and (2) How does the relationship between industry and academia impact the research process? Our findings reveal diverse approaches to human detection, ranging from basic obstacle avoidance to advanced systems that differentiate among classes of humans. We suggest that robotic system design overall and human detection in particular are influenced by whether researchers use a framework of safety or sociality, how they approach building complex systems, and how they develop metrics for success. Additionally, we highlight the gaps and synergies between industry and academic research, particularly regarding commercial readiness and the incorporation of human-robot interaction (HRI) principles into robotic development. This study underscores the importance of addressing the complexities of social navigation in real-world settings and suggests that strengthening avenues of communication between industry and academia will help to shape a sustainable role for robots in the physical and social world.

## 1 Introduction and related work

Mobile robots are rapidly moving from controlled settings into human-populated environments, where detecting and responding to people is central to safe and productive operation (Gartner, 2023; Rubio et al., 2019). Applications span logistics, surveillance, maintenance, transportation, and service settings (Jahn et al., 2020; Sharma et al., 2023), but development across these sectors shares common challenges, particularly around human detection and interaction (Mavrogiannis et al., 2023), integration of cutting-edge research, and commercial maturation (Carlsson et al., 2002; Hekkert et al., 2007; Matarić, 2018). While sensing, navigation, and mobility capabilities are evolving rapidly (Patle et al., 2019), enhancing robots’ abilities to detect human movement and gestures is critical for navigating complex settings facilitate co-deployment and collaboration (Navarro et al., 2021). This broader scope places human detection at the heart of human-robot interaction (HRI) as a field that integrates technical innovation with the social, economic, and governance contexts in which robots are designed and deployed (Stam, 2015; Hannigan et al., 2022; Robey et al., 2001; Kuhlmann et al., 2019; Serrano and Fischer, 2007).

HRI has expanded as an interdisciplinary field integrating AI, robotics engineering, cognitive science, and policy and design thinking (Sinapov et al., 2024) applied across domains such as healthcare (Brooks et al., 2024), farming (Cila et al., 2024), education (Byeon et al., 2024), hazardous environments (Pérez-D’Arpino et al., 2024), and industrial automation (Mukherjee et al., 2022). Robots in social spaces impact not only designated “users” but also bystanders (Dobrosovestnova and Weiss, 2024) who may be incidentally co-present (Rosenthal-von der Pütten et al., 2020) or engaged in unplanned interaction with the robot. This work emphasizes the complexities of trust, accessibility, and context-specific design (Mckenna et al., 2024), particularly as robots transition from experimental prototypes to practical tools in real-world environments (Joshi, 2023). Additionally, this literature increasingly underscores the importance of incorporating the insights of non-technical stakeholders (Joshi, 2023) to ensure commercial robots are not only technically robust but also usable and aligned with human and societal needs (Cila et al., 2024; Wang et al., 2019). Effective interaction however depends not only on social design principles but also on the robot’s perceptual and sensing capacities.

Effective environmental sensing and object detection are crucial in HRI as they enable robots to accurately perceive and interpret human presence, actions, and the surrounding environment (Bonci et al., 2021). Technical advances in sensing and computation, including low-cost high-resolution sensors (Robinson et al., 2023), improved processor efficiency (Chen et al., 2022; Cervera, 2020), and advances in classical optimization methods (Banisetty et al., 2021), machine learning methods (Samsani and Muhammad, 2021), or hybrid learning systems (Gil et al., 2021), have expanded possibilities for human-specific detection and, relatedly, deployment in complex or populated environments. Yet progress in academic research often struggles to scale into commercial systems (Gil et al., 2023), where inconsistent metrics create obstacles for comparison across navigation approaches and complicate evaluation of safety and usability (Mavrogiannis et al., 2023).

A further tension arises between the academic and industrial settings in which robot innovation is occurring under different resource and economic constraints. Academic research is often driven by novelty, controlled evaluation, and publication incentives, while industry emphasizes robust reliability, scalability under cost constraints, and market positioning (United Nation, 2015). Our findings suggest that while person detection may appear solved in controlled environments, it remains unevenly deployed in commercial settings. A small but growing, body of HRI literature, therefore, has turned to industry perspectives to understand how robots are being designed and built in practice. Tsoi et al. (2024) found that roboticists ranked collision avoidance and intimate distance violations as their highest priorities in social robot navigation. Dobrosovestnova and Weiss (2024) showed how commercial robot development draws on feedback from controlled laboratory testing as well as from feedback from deployment in the field, including planned interaction with users and unplanned encounters with bystanders.

Bridging these divergent settings requires understanding person detection not only as a technical capability but as a socio-technical problem that sits at the intersection of safety, sociality, and performance (Kuhlmann et al., 2019; Salazar and Russi-Vigoya, 2021; Bernstein et al., 2022). In industrial contexts, person detection is often framed through a logic of safety, or the imperative to prevent contact, ensure reliability, and maintain predictable operation within regulatory bounds (Mavrogiannis et al., 2023). In contrast, research on social navigation pursues a logic of sociality, in which robots must perceive, interpret, and sometimes anticipate human behavior to inhabit social environments (de Graaf and Malle, 2019; Thompson et al., 2025). These two logics coexist uneasily: emphasizing safety can limit interaction, while emphasizing sociality can introduce risk and uncertainty. From the perspective of innovation-systems and Technology Readiness Level (TRL) frameworks, this tension marks a transition from lab or pilot-grade safety to more robust social-commercial readiness (Östlund et al., 2023; Eriksson and Music, 2021), which can entail functional reliability alongside product integration into organizational, regulatory, and human-interaction contexts (Yfanti and Sakkas, 2024; Salvador-Carulla et al., 2024). By examining how engineers navigate this continuum, we situate person detection as a challenge of socio-technical coordination, where the development of a robotics system is characterized, in part, by how it mediates human–robot relations. This paper extends that line of work by examining how commercial engineers conceptualize human detection and the relationship between academic research and industrial practice. Drawing on qualitative interviews with sixteen roboticists with industry experience in the field of social mobile robots, we asked two research questions: (Gartner, 2023) What approaches to person detection are used in practice in industry, and (Rubio et al., 2019) how does the industry-academia shape the research process? Our findings suggest that engineers diverge in how they prioritize human identification for safety and performance, how contextual and environmental clues are incorporated into training social behavior, and when “acting socially” is prioritized over optimized robotic function in isolation. In discussing industry’s message to academia, we highlight the gaps as well as the complementary roles for disparate actors in the robotics ecosystem, noting how industry professionals conceptualize commercial readiness, identify metrics for social robotic success measurement, and understand the field of human-robot interaction in the context of developing commercially-deployable robots.

## 2 Methodology

We conducted semi-structured interviews with 16 industry professionals involved in building, designing, or deploying robots that interact with humans—a group we term “social navigation roboticists.” To align with our research focus on industry–academia gaps, all participants had current or recent industry experience.

Interviews followed an 11-question semi-structured protocol, developed to incorporate interdisciplinary concerns through feedback from university faculty in computer science, engineering, and public policy. Topics included robot use cases, navigation systems, perception and sensing, modeling approaches (classical and data-driven modeling), product development feedback channels, and challenges or bottlenecks in research, design, and commercialization, and views on the most pressing industrial challenges they felt deserved greater attention from academic researchers. Clarifying follow-ups were added as needed; the full protocol is available upon request. Interviews lasted 45–60 min, were conducted via video call, recorded with consent, and anonymized under a Rutgers University IRB-approved protocol Pro2020001632.

### 2.1 Participants

Participants were recruited through LinkedIn, faculty and university networks, professional associations, and referrals from prior contacts. Approximately 25 professionals were invited, and sixteen agreed to participate. Because this study targeted a small population of senior engineers and technical leads in commercial robotics, participant availability, rather than data saturation, was the principal limiting factor in recruitment, a common characteristic of expert-interview research in emergent industries. Eligible respondents were required to be active industry professionals with at least 5 years of experience in the field. Despite outreach to a gender-diverse pool, all participants were male.

To capture diversity across robot form factors and human-environment contexts, we sought participants from multiple application domains, including self-driving vehicles, warehousing and logistics, consumer electronics, inspection, and defense. Representation was necessarily uneven, reflecting our professional network and the present concentration of active commercial deployments in logistics and autonomous-vehicle sectors. The participants had an average of 12 years of industry experience ( σ = 7). Respondents included five directors or managers, seven senior or principal engineers or scientists, one professor, and three CTOs. Six respondents had experience in academia before entering industry. Table 1 summarizes the characteristics of participants in this study. Some participants had experience in more than one sector. They are included in the count for all of the sectors in which they have worked. In addition to documenting participants’ backgrounds, we also recorded the types of robotic systems discussed during interviews. Across the sixteen participants, these interviews covered twelve unique commercial robots spanning self-driving vehicles, warehouse transport robots, consumer service robots, and quadrupedal inspection platforms. These systems varied in form factor, operating environment, navigation constraints, and person-detection capabilities. To protect confidentiality, robot descriptions are presented only at this functional level of abstraction. Table 3 provides an anonymized overview of the robot systems represented in the study, along with interview data detailing at a high-level sensing, navigation, and person detection approaches.

**TABLE 1:** Participant characteristics.

Characteristic	Count
Total participants	16
Average years of industry experience	12 ( σ = 7)
Seniority	5 directors/managers
	7 senior/principal engineers/scientists
	3 CTOs
	1 professor
Prior academic experience	6
Sectors represented*	10 Warehousing/Logistics
	5 Consumer electronics
	1 Defense
	2 Inspection

*Some participants work in multiple sectors, so counts may overlap.

### 2.2 Data analysis

As a cross-disciplinary team of researchers, we combined our engineering and social science backgrounds to investigate how mobile robots are being embedded in social spaces. Both researchers participated in each interview. Interviews were recorded and transcribed for qualitative coding. Each author open-coded a first round independently, with codes iteratively refined to establish an agreed-upon codebook (Pratt, 2023). Using an aligned codebook, we performed a second round of coding. Next, we identified clusters of codes and developed conceptual categories, again informed by concepts derived from the literature (Marshall et al., 2021). Each author wrote thematic memos to summarize key ideas signified by the open-codes. These categories were used to inform emergent themes, again informed by the prior literature review (Marshall et al., 2021). Coding reliability was ensured through joint reconciliation of code assignments and regular cross-checking between authors rather than quantitative scoring, consistent with qualitative best practice for expert-interview studies. A summary of the final codebook, including five major code categories and their emergent themes, is presented in Table 2. To complement the qualitative coding, we also conducted quantitative frequency analysis of thematic trends across participants.

**TABLE 2:** Partial codebook.

Major code	Sub-code & keywords	Emergent themes
Person detection	Safety, detection range, false negatives, lidar, thermal, multimodal sensing, physical barriers, context, density of people	Environmental and technical constraints shaping perception; performance trade-offs
Human–environment context	Warehouse, self-driving, mobile, isolation from humans, rules of the road, carrying, challenging spaces	Varieties of human proximity/interaction; adaptation and design for social environments
Reliability and performance	Rigor, super reliability, generalizability, massive datasets, data flywheel, connected robot, cloud	Commercial readiness; reliability at scale; data production and use
Safety vs. sociality framework	Deference, interaction rules, motion planning, perceptual challenges, people	Balancing avoidance and engagement; avoidance and safety versus interaction & complexity
Message to academia	Investigate whole/complex problems, certification, resource constraints, lack of ambition/imagination	Industry’s call for realism and robustness in academic HRI; gap between controlled research and deployment complexity

## 3 Results

Through these interviews, we sought to develop a detailed and nuanced view of how challenges related to human-robot interaction, and social navigation in particular, are considered in industry. Industry professionals who participated in this study offered grounded and pragmatic perspectives as well as conceptual and theoretical views on best practices. Data on approaches to person detection in robotics yielded four important themes: (1) person/object differentiation; (2) context and environment as integral to detection; (3) use-case as an optimizing framework; and (4) how robots can learn to act socially in complex human environments. Responses to industry’s message to academia focused on three key themes: (1) commercial readiness; (2) emergent problems at scale; and (3) incorporating HRI into robotics development.

### 3.1 Person detection

#### 3.1.1 Person detection and human-object differentiation

**FIGURE 1:**
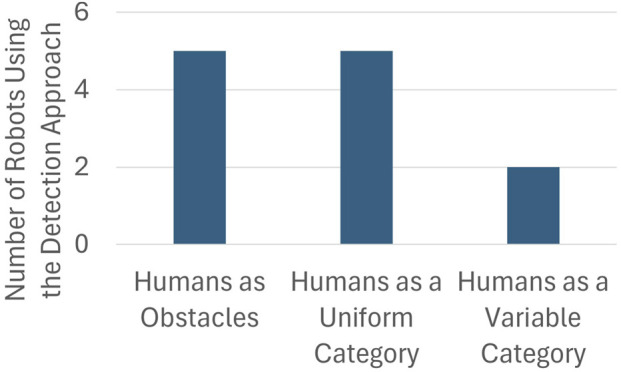
Approaches to person detection used in the social mobile robot industry.

A central question to our study was: how are people represented in a robot’s planning environment? Respondents offered three broad approaches to human detection: first, systems in which people are not differentiated from other obstacles (humans as obstacles); second, systems in which people are detected as a distinct but uniform category (humans as a uniform category); and third, systems in which robots not only distinguish people from nonhuman obstacles but also differentiate among types of humans (humans as a variable category). Twelve unique robots were worked on by the participants in this study. Although this is far from a fully representative sample of the commercial space, it offers a telling snapshot of the current state of person detection in robots currently being deployed. All of the robotic systems represented in this study are custom-built by the companies that the participants work for. We ensure the anonymity of our participants by withholding identifying details about the robots that they are building, however Table 3 provides some context about the robotic systems being developed by the participants in this study. To better understand the current state of person detection capabilities, we identified the function of the technology used on each of the twelve robots. These results, illustrated in Figure 1, show that five robots worked on by participants currently have no person detection capability, another five robots have uniform person detection, while two robots can distinguish between different types of people.

**TABLE 3:** Robot categories and characteristics.

Robot category (industry sector)	Number of robots	Primary operating environment	Navigation constraints	Sensing technologies	Person-detection approach
Passenger-transport autonomous vehicles (self-driving)	2	Unstructured public roads	Multi-agent interaction; occlusions; long-range behavioral prediction	Lidar, radar, RGB cameras, depth sensors, IMU	Differentiated human classes
Freight/yard autonomous vehicles (logistics and depots)	2	Depot perimeters; outdoor industrial yards	High-speed interactions; sparse agents; variable lighting	Lidar, radar, RGB cameras; outdoor-tolerant sensing	Uniform human detection
Warehouse rack-moving robots (logistics)	3	Highly structured indoor layouts	Narrow aisles; predictable flows; sightline constraints	2D lidar, RGB/depth cameras, IMU, odometry	Typically none → uniform human detection
Warehouse pallet/case movers (logistics)	2	Semi-structured mixed-traffic warehouse zones	Blind corners; human–robot intersections	2D/3D lidar, RGB cameras, depth sensing	Uniform human detection
Consumer cleaning/service robots (Consumer electronics)	2	Structured domestic or segmented commercial interiors	Clutter; irregular obstacles; short-range sensing	RGB cameras, depth/light sensors, basic proximity sensors	Typically none
Quadruped inspection/tactical robots (Defense, Inspection)	3	Unstructured industrial, hazardous, or outdoor environments	Terrain variation; low visibility; uneven surfaces	RGB cameras, depth/stereo, thermal imaging, IMU, proprioception	Typically none

First, some robots in development or in use rely mainly on low-level safety systems designed to prevent robots from colliding with obstacles, including people. These systems do not have to distinguish people from other obstacles to trigger safety behaviors, such as stopping. As one respondent put it, “at the moment, we do not differentiate [between] human and object. If there is someone or something in front of the robot, it will stop and wait for the obstacle to move away. And after a certain amount of time, it will find another path to its target.” Some respondents said that person detection was not necessary for safety; or, as one engineer elaborated, “right now, our main focus is to be safe. And to be safe, we just need to avoid all types of obstacles. So, that is why we do not focus on differentiating humans from other things.” Robots that are in this category include a robot vacuum, two quadrupeds, a humanoid, and one that operates in warehouse settings.

In other cases, specialized human detection is already standard and considered necessary for safety. Industrial robots that share space with other agents, one engineer said, need to be able to classify moving objects so as to “unbound their behavior more accurately, accounting for their capacity to move.” One respondent said, “We detect people and treat them specially. People get a right-of-way affordance, and at the bottom layer of the system, the reactive system causes the vehicle to freeze if an agent gets too close.” Here, the respondent points out that specialized human detection systems are layered on low-level safety systems that provide redundancy. Representative technologies in this category include two other warehouse robots, another quadruped, a person-following robot, and a self-driving vehicle.

Finally, some robots being produced at scale not only recognize persons but distinguish among classes of humans. Integrating “a semantic level of person detection,” as one respondent put it, allows robots to distinguish “a police officer from a construction worker” and to know “which people have the right of way or you have to stop for.” One respondent said, “There are all sorts of designations and modifiers for pedestrians and other agents, like crossing guards, emergency vehicles, or cyclists.” Another engineer said they focused on unusual cases in which a person may “move in un-person-like ways,” such as when “on a scooter or on a skateboard [a person] has very different dynamics than a typical pedestrian.” Self-driving cars are a hot spot of focus on human classification so that, as one engineer put it, autonomous vehicles can recognize the relative “vulnerability of a road user,” including “subdivisions within the class of ‘people,’ like children who are even less predictable than adults and harder to see.” That engineer said it was important for autonomous vehicles (AVs) to recognize, for example, that “people inside a vehicle are clearly more protected than people not inside a vehicle.” Both of the robots represented in this category are self-driving vehicles.

Though most engineers said human identification was a key research question, most framed it as an area of future development rather than standard current practice. Human detection appears to be standard practice for self-driving cars and some other AVs, but many commercial robots we learned about in this study do not yet differentiate people from other objects. The range, complexity, and unpredictability of human behaviors continue to make person-detection such a challenge that one respondent who worked in logistics and warehousing said “we’re not differentiating between types of people yet,” while another whose multipurpose surveillance and safety robot is already in use agreed human detection was not yet incorporated into their robot’s perception capabilities, but “that’s very much an area of research right now.”

#### 3.1.2 Person detection: contextual clues and environmental factors

Person-detection is informed by context and several engineers echoed “the necessity of perceiving different types of contexts” so robots can, for example, interpret and plan around human behavior. Several respondents argued individual and group behavior must be modeled differently because, as one said: “There’s a big difference between one person and a couple of people walking together.” Another agreed that crowds produce emergent behaviors not displayed by individuals, like when “a stadium is letting out, it is probably not useful to look at any individual person and ask, what are they going to do? The crowd dynamics matter more than any individual there.” Moreover, behavior flows from groups, but also environmental context; as one engineer said, “it matters how many people are in the context. If the robot is on the street, that’s a lot of people, but a household and a workspace are shared spaces with a limited number of people.”

Sightlines and spatial scale are also important environmental factors. One roboticist who oversaw the development of robots for large logistical settings remarked how sensor constraints meant that robots would not be able to detect or respond to humans especially at a distance:

“Range is a constraining factor. RGBD sensors for indoor use cannot detect people far away. But, people can engage at a distance; and the range that people react to each other is highly context dependent. In a crowded or cluttered space, interaction might happen when people are close. But in wide open situation, people respond to each other from far away– and I’m not talking just about active communication-like speech or gesture, it’s more that people will nudge their trajectory to one side or the other as they’re walking to make it clear which side they intend to pass on.”

Another engineer in warehousing logistics said situations with limited physical sight lines may limit a robotic sensor so a machine “sees” only part of a human–a head or body part behind a stack of boxes–resulting in a “lot of false negative detections.” Taking environmental constraints into account when building and selecting sensors is therefore critical for accurate human detection.

### 3.2 Optimization versus integration

A key area of disagreement among respondents centered on whether sharing spaces with humans is the optimal use for robots and, if so, how robots should be taught to navigate complex environments. Some engineers argued that building for robot-only environments optimizes efficient, safe, and productive use cases. These respondents argued that instead of beginning with the question of how to refine human-detection, we should ask whether it is necessary or optimal for robots to deploy in spaces shared by humans. The degree of control that engineers have over the robot’s end-use environment (a warehouse compared to a pedestrian street) affects this decision. Two engineers in logistics and warehousing argued that robots working in isolation can be optimized for productivity and this approach will continue to have industry applications in the coming years. As one said, “ultimately, this industry will remove pedestrians from the logistics operating area, not just for safety, but for performance.” Where space is not an issue, another argued, it makes sense to simply “segregate pedestrians to the piece-picking area, and the robots to the case-picking area.” Another respondent said they had worked at companies that took “both extremes,” and noted that “in a warehouse environment, the thinking around safety is very much ‘we’ll slap a fence around it [and] nobody will interact with the robot.”’ But, the same engineer said, that only works for some types of industrial robots, “obviously you cannot do that with self-driving cars.”

Engineers who advocated for integrating robots into human environments were also internally divided about best practices. Some said humans should be part of robot development from the beginning while others said robot integration must be approached through a stepwise development pathway.

Those in favor of ramping up complexity suggested beginning with simple deployment scenarios (robots isolated from or trained to cease motion around humans) and moving toward sophisticated use cases in which robots could work alongside humans or be integrated into populated environments. One engineer said development generally begins with perfecting controlled environments and progressing from there because “interacting with humans is really hard. Generally, in the world of robotics and products, the easiest thing to deploy is a robot that works in isolation. Because you could work on that system, perfect it, send it to the customer and then it just works and no one messes with it. So that’s the easiest thing and where most products start.” Another engineer said they thought of person recognition and safety “in a hierarchical sense,” where “at a lower level you have dynamic obstacle representation where humans are not something different than any other dynamic obstacle,” but building on that, “we need at the top level some way to identify different classes of things in the environment, and people are a very important class.”

Building human recognition into an increasingly complex perception stack can also reflect regulatory requirements. In some cases, regulation shaped the development process because, as one engineer said, “if you want to release a product into an environment where it is no longer physically separated from people, you have to pass safety certifications, which is expensive and hard.” This respondent added: “at this point, the bar is: do not run into people, do not kill people,” but then if “you want to go to the next step of being able to interact with people closely, that’s more challenging” because safety and interaction have to be more tightly integrated. As the same respondent pointed out, if the robot is “meant to interact with a person, you cannot just say, hey, if there’s a person within a 1 m radius of the robot, just stop. You need to enable moving in close proximity with people. And that’s really hard.”

Other engineers approached the problem differently, with some arguing that rather than building complexity ladders, you should start with the most realistic–even if complex–use case for robots. One said, most “economic activity happens around people,” while others pointed out that there is no point in optimizing self-driving cars for isolated controlled environments; instead, one engineer stated, “it is obvious that from the get-go you need to handle humans appropriately.”

Intended use cases also shape a company’s approach to person detection. For robots engaged in a following task, one respondent pointed out, recognizing the person is the first goal. Or, if human interaction will be critical to a robot’s use, then teaching “non-experts to safely operate” a robot is critical. Importantly, interface design is key so users can unlock a robot’s potential uses, or what one engineer called its “taskability,” especially if a robot has many capabilities, from, “taking pictures and carrying payloads, to thermal scanning and vibration sensing.” In these cases a commercial robot engineer said, “what is as challenging [as safety] is taskability or how you communicate to the robot what you want it to do.” This problem can be solved with simple interfaces–domestic cleaning robots with a single button–or, as several respondents suggested, through the growing capacity of large language models to allow natural-language direction. However, the challenge remains that “the more capabilities your robot has, the more things it can do, the harder it becomes to let a non-expert user program the robot in a flexible way.” Intuitive interfaces and making clear through design how a user can direct a robot was stressed by our respondents as essential for machines designed to participate in the human world. As one engineer concluded: from an engineering perspective, “it may be complicated for the robot to do what it is supposed to do, but it must be easy for the human to ask it to do that task.”

#### 3.2.1 Acting socially and training for human complexity

Alongside the challenges of person detection and safety optimization, respondents highlighted the steep learning curve of teaching robots how to “act socially.” For robots to interact with humans, not just freeze or avoid them, engineers have to think about how robots behave around people. One said they thought about a “robot attitude,” explaining, “robots working in environments shared with human beings need to be respectful and work around what humans are doing. Only then can we talk about whether robots can be helpful.” Another said: “There is a negotiation between how much you want to approach the person and how much to avoid them.” Neither inert nor interrupting robots are helpful and well-integrated robots. Cleaning robots, one respondent said, ideally recognize when users are “playing the piano or having a meeting.” More broadly, “there need to be mechanisms for robots to read a situation and then either end a job early or assess if there are still spaces robots can claim without disturbance to the user.”

Integrating robots into complex environments without endangering or inconveniencing humans requires, as one roboticist in the self-driving space said, huge training datasets that include fringe scenarios. While the AV industry is now big enough so some companies develop niche expertise, like “autonomous trucking companies that focus on straight land driving and fuel economy,” while ride-hailing companies need to “from the get-go, focus on environments with lots of complex interactions.” Thinking about both “the big picture and how a technology and a system will be scaled” depends on developing the technology for the right framework.

Finally, professionals in commercial robotics often reminded us that human detection and human behavior models are business decisions. Respondents spoke about building trust with customers and public representatives. “We try really hard to be good citizens,” one engineer said, and “trust, probably at the most local level, is critical.” This can rely on a “tight feedback loop [with] local government regulators” among other stakeholders. Central to building trust, this engineer argued, is demonstrating “this thing actually works.” For some companies, being responsive to customer needs meant setting aside, for now, specialized human detection to focus on, for example, tailoring a robot to warehouse settings or specialized tasks like inspecting offshore drilling rigs where human interaction was minimal. We keep in mind, one engineer said, that “the robots are there for a purpose and we want to make sure they are valuable, for example by improving efficiency, having a reliable system, and integrating well with their facilities.”

“At the end of the day,” another respondent said, “customers do not care how the technology works, they just want it to do a job, do it efficiently and be cost effective and safe.” Therefore, this engineer said, experts in the field have to decide what is safe and optimal. But, they added, while “it does not matter what technology you put in it, I think we’re entering a new era where products will be more software-defined so that we can ship a piece of hardware and then update its software” as navigation and perception best practices change over time.

### 3.3 Messages from industry to academia

The second research question investigated the relationship between industry and academia impact the research process. We aimed to understand what challenges and opportunities the academic research community should be focused on from an industry perspective. To this end participants were asked: “what message would you share with the academic community?” Respondents gave a broad range of answers to this open-ended question. Many prefaced their opinions by saying they were not experts on the current state of academic research, a valuable datapoint in and of itself. We grouped their responses into three broad categories, discussed below.

#### 3.3.1 Commercial readiness

Commercial Readiness encompasses ideas of technological maturity and whether systems meet current safety standards. Respondents contrasted the freedom to experiment and fail in academic settings with the bar of exhaustive reliability in industry settings. One noted that “in academia you can try out an idea and you do not have to make that idea successful. You can try something out, write a paper, done.” By contrast, in industry, another explained, you fixate on “how long does a robot run before something breaks and that needs to be in the hundreds or thousands of hours.” Engineers “focus on reliability in every part of the stack.” Performance, another said, “has to be really robust, really work, and not just be a fancy algorithm.” Respondents emphasized long-term reliability over novel solutions, one saying that commercial success was about “high quality software, high quality process speeds, high quality evaluation.” One respondent used the Technology Readiness Level, or TRL, scale, to explain differences in academic and industry priorities. Originally developed by NASA to describe the flight-readiness of its systems, the TRL scale goes from level 1 (basic research) to level 9 (full scale deployment). In a commercial setting, this respondent said:

“We’re trying to take something that has been shown to work in a lab and take it to a level of polish and reliability that it can be deployed to a customer. The performance and reliability requirements, even for the research group here, are way higher than in academia. If you know what Technology Readiness Level is, it’s like that concept.”

Alongside dependability, respondents said “the most important thing [for commercial robots] is safety.” Safety was connected to, as discussed above, environmental control and complexity. Fully-known environments can be certified as safe with the addition of light curtains or other safety-rated sensors, which would be insufficient for unmapped environments. Safety was key for both autonomous and non-autonomous robots. One researcher noted that a human-machine interface (HMI) might be designed one way for a research setting so that “if you click the wrong button, bad things can happen,” but differently if “you are going to give a tablet to a customer, [where] it is got to be really simple and safe.” Several respondents noted that safety certifications are time challenging, but others raised more structural challenges about making a robot ready for industrial deployment. One asked: “how can we come up with better mechanisms to implement safe systems in the lab that are certifiable?” These standards could guide social robot development in both academic and industry settings, while improving safety standards across the board.

#### 3.3.2 Emergent problems at scale

The second theme in our data about this question was how problems emerged at scale. Respondents outlined how critical industry challenges are often not identifiable or solvable in small lab systems, and require commercial deployment, integration with realistically complex systems, and stress-testing at large scale to emerge. Most engineers emphasized it was not the job of academics to build systems ready for industrial scale deployment–and that academia and industry played complementary roles–others said academics could do more to confront challenges like reliability that tend to separate academic and industrial solutions.

Respondents described how academic research can fall short of solving challenging problems in social navigation not due to a lack of creativity, skill, or effort, but because these problems only emerge when working at the scale and complexity of a real-world system. As one respondent said, “in terms of social navigation, I think scale is probably the big thing that is missing in academic research.” Another added that though academics are understandably focused on small lab contexts, they need to “think about the scale of a warehouse [and] a solution that will work over a football field densely cluttered with stuff.” What may appear to an academic researcher to be a solution, does not necessarily provide “ideas that are rigorous at scale.” And yet, one respondent said, this standard might be “unfair” because in academia “there are problems that you simply cannot observe unless you operate at scale.”

Additionally, some solutions are only feasible using industry-scale infrastructure, such as access to huge computational resources. Many respondents talked about the inverse industry/academia data challenges. While some companies make datasets (stripped of identifying information) public, the amount of data collected by a company operating at scale dwarfs what academic research groups can access. One respondent put it simply, “the amount of data that most of the companies have, it is really a goldmine.” Another observed that “in academia everyone is trying to get more data and our problem [in industry] is that we have so much data that we have to throw 99% of it away because it is not worth the cost to store it.” Another respondent described how once a robot is deployed, it will generate huge amounts of data through its commercial use. Information harvested from this “data flywheel” can be used to refine the product, a course academics do not have the option of pursuing.

Finally, respondents pointed out that emergent problems demand examining an entire system rather than a single issue. While industry engineers deal with real-world complexity, academics “try to simplify the problem to get to a paper” and fail to “look at the problem as a whole and solve it.” Instead, this engineer suggested, academics should “try to formulate research topics that are more realistic for real-world application” by taking on “the clutter and messiness of the real world.” These experiments might tackle “hardware challenges, like sealing sensors properly for navigating in a muddy environment” or move away from “assuming a 2D environment, like most work in mobile navigation, which is really not the case when you go outside and try to go over hills.” Despite agreement that academics were focusing on the performance or complexity conditions required in industry settings, some respondents understood this as an unrealistic bar for lab researchers and that academic researchers and industrial developers held fundamentally different roles; as one respondent reflected, “putting it all into one soup … there’s a lot going on in there. I do not know that I see academia really grappling with that, but I also do not know if that’s actually where academia should be. I have mixed feelings about that overall.”

#### 3.3.3 The role of HRI

Finally, respondents spoke about the perception of the field of HRI in industry, and a ‘wish list’ of research topics academics looking to contribute to the HRI community might address. One respondent shared a view echoed by others that “a lot of people in industry are ignorant about HRI or its importance. I think a lot of them brush it off as being like UX, or the sort of the thing we can just do to add a little polish at the end, and we’ve solved HRI. There’s a very naive approach to it generally.” This first-person account highlights the need for the HRI community to consider how to effectively convey the importance of its work to decision makers in industry that shape how robots out in the world are being built. This respondent noted that some in industry misconstrue HRI as “just part of safety,” even though safety, as discussed above, has a complicated relationship to sociality.

One way to demonstrate the value of HRI research is to solve problems that are meaningful to those in industry. This agenda setting could happen more readily, as one respondent said, “if academia could step into the industrial world before formulating research problems, they could find more valuable problems to work on, or inspiration for better ways to solve problems.” Across our respondents, we identified several broad areas industry experts felt would be desirable for academics to address (the italicized themes below reflect our distillation of the qualitative data).

#### 3.3.4 Incorporating HRI into robot development

Industry respondents highlighted the importance of what we term a hierarchy of deference, or how robots identify types of people, distinguish among them, and define the rules of deference. One said, “I would love to see more detailed research on the hierarchy of deference of social navigation… I think there’s a recognition problem that I’ve never seen anyone try to solve from a social standpoint. Much less how to actually reason about it.” Industry professionals also pointed out that more research should be done into predefined rules for robots, or the social norms robots should adhere to, ignore, or redefine. As one respondent said, it would be good to know “what literature a designer can turn to know how to drop a robot into a setting that has a pre-existing set of rules and responsibilities.”

#### 3.3.5 Addressing industry challenges and standards

**FIGURE 2:**
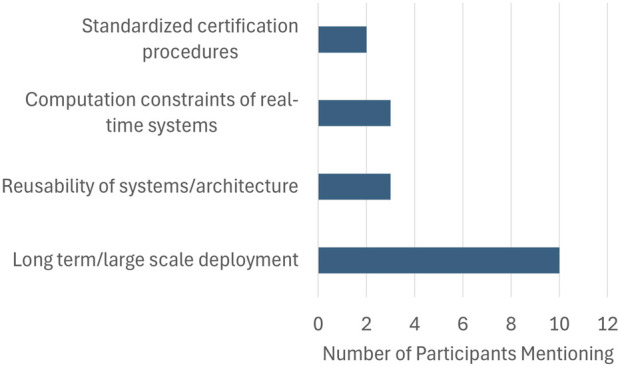
Research needs repeatedly mentioned by participants.

Industry experts identified challenges in several areas they felt deserved more attention in academia. Figure 2 highlights those challenges that were mentioned by multiple respondents. Long-term robot deployment, or what methods and designs can prepare a prototype for deployment at scale, came up much more frequently than any others, as illustrated in Figure 2. One respondent said, “a lab of three people that keeps 100 robots running would have done something interesting.” This need and novelty is punctuated by the observation that much recent research in this area has relied on testing in simulation (Eiffert et al., 2020), which removes many important factors from the tests. However, researchers can hardly be blamed for this, as long-term research projects have higher risks with limited additional benefits (Foster et al., 2015). In addition to long-term deployment, experts also focused on “reusability,” or how researchers can develop system architectures that can be repurposed in different contexts. However, as Figure 2 shows that this and subsequent challenges occurred far less frequently than the long-term deployment challenge. Another respondent said researchers should ask themselves: “How does the structure fit together and what parts are reusable, what parts can be redeployed to solve different problems or other areas of the system?” Other respondents said academics take greater account of the compute-constraints of real-time systems and build experiments in frameworks that are realistic about the computational power available for a commercial robot. One respondent said, “It is easy to throw something in a data center and allow infinite compute or many GPUs. This is useful for thinking ‘here’s how we would like to solve a problem,’ I think that’s different from ‘here’s how we can solve the problem.”’ Finally, experts pointed to the value of standardized certification procedures for commercial settings. As one respondent said, “Look at the current certification procedures that are out there and ask how we can improve them and assess them.” Standardized procedures can reduce risk, telling developers exactly what is allowed, and therefore constraining, but also facilitating the research process by removing uncertainty.

The results reveal how engineers approach human detection, integration, and collaboration not as isolated design questions but as interdependent logics within an innovation system. Across themes, three mechanisms appeared repeatedly: (Gartner, 2023) the conceptual starting point of design (safety, sociality, or performance); (Rubio et al., 2019) organizational constraints, such as certification requirements and customer interaction and expectations; (Jahn et al., 2020) pathways toward commercial readiness require balancing these priorities under real-world uncertainty. The following discussion draws these threads together to synthesize how person detection functions as a technical and socio-technical coordination problem, linking laboratory development to commercial deployment.

## 4 Discussion

### 4.1 A framework of safety or sociality

Our findings suggest that the conceptual framework or starting point for robotic design is critical for understanding development pathways, particularly for human recognition. This starting framework shapes what problems engineers identify and try to solve. We suggest that industry experts broadly outlined three starting points for robot design: 1. Safety, 2. Sociality, and 3. Technical performance requirements.

Though goals implied by these frameworks may be complementary, we suggest there are also points of tension. For example, beginning with safety as an absolute priority may require robots simply come to a halt around humans and only operate once humans are at a safe distance. This kind of behavior is not social in an interactive or collaborative sense and may, as several respondents said, create awkward or even obstructive behavior, requiring humans to go around, accommodate, or ignore robots in their environments. Safety therefore can come at the expense of social behaviors. However, beginning with sociality as a goal may require engineers to prioritize interaction and continued work or movement in close proximity to humans over absolute safety. Yet, while some industry respondents said robot deployment in human environments was a clear priority in their businesses, many reported that they still did not begin the design process with the idea of social interaction, but started with controlled, often isolated environments designed to optimize safety and technical performance in the forefront, and only then scaled a ladder of complexity (see below).

Finally, the framework of technical performance reflected a commercial bottom line of meeting customer expectations. Robots must perform the tasks they are marketed as being able to do, and do so at a level of high performance and reliability. Technical performance therefore can trump sociality (imagine a delivery robot that people enjoy interacting with on the sidewalk, but never reaches its destination). The tension that emerges reflects efficiency as well as proficiency. Though a team may have the technical acumen to build a socially cognizant robotic system, these additional requirements may slow production or increase costs. Companies therefore weigh safety, performance, costs, and customer feedback in determining what capabilities are necessary for a viable product. Despite these costs, when ‘socially cognizant robots’ are part of the company’s use case, then sociality may be part of technical performance and necessary for a viable product.

The findings can be understood as mapping how engineers position their systems along a safety–sociality continuum In some systems, safety may dominate, with robots designed to simply avoid humans entirely; in other systems, sociality is integral, with robots required to engage or negotiate close proximity in shared space (Mavrogiannis et al., 2023; Bartneck and Moltchanova, 2020). This framing aligns with recent calls in the HRI and innovation-systems literature to treat commercial readiness as a process of socio-technical integration as well as technical improvement (Kuhlmann et al., 2019; Östlund et al., 2023; Eriksson and Music, 2021). Readiness, in this sense, entails reconciling performance reliability with social compatibility (Bernstein et al., 2022). Viewing industry practice through this lens clarifies why many engineers described person detection as “solved” in principle but nevertheless a persistent challenge in deployment since what is technically solved remains socially unready.

### 4.2 Complexity ladder

Industry experts were divided over whether robotic systems should be optimized for simple environments first or confronted with complexity even at the earliest stages. These decisions have far-reaching implications for the development workflow, including the kinds of expertise needed on a team, as well as hardware and software design choices. This question is closely linked to that of human-detection. While some roboticists did not think human-detection was key to safety, everyone agreed it was an area of active research if not current use. For some roboticists it is already a priority in their day-to-day work and central to their robots’ functionality. These companies tend to begin with rather than build complexity. Finally, some respondents pointed out that while their robots currently do not distinguish humans from other obstacles, they are built on systems with the capacity to incorporate that in the future, or scale the complexity ladder. As one respondent stated, “we are not differentiating between different types of people right now, that is very much an area of research,” and another said, “the answer right now is ‘no,’ but the intent is ‘yes.’ We have machine learning methods to label objects and though we can label ‘people,’ the robots are not doing any particular behavior in response to that.” Or as another put it, “as of now we do not detect people, but it will probably be part of development in the future.”

### 4.3 Metrics for success

Technology and product design rely on establishing metrics for success, or the measurements used to decide if a project is on track, a system has achieved its goals, or a product is successful. Traditional success metrics include quantified areas like technical performance, budget, and schedule. Common robot technical performance metrics include power consumption, process speed, and mean time between failures. While some HRI-related metrics exist, they tend to focus on human-robot proximity. There is a need for supplemental metrics to guide and assess social robotic system design. Establishing such metrics is challenging given an “absence of a uniformly agreed upon evaluation standard” for robots in social navigation settings (Mavrogiannis et al., 2023). To develop social performance or integration metrics, typical measurements mentioned above can be supplemented by social navigation-focused metrics like collision rate, path irregularity, acceptable buffers or acceleration rate, and others. Any chosen metric may have downsides, such as lack of representativeness or challenges in evaluation, a commitment to include any measure of sociality advances the aim of building more socially acceptable robots. How safety, sociality, and commercial readiness are weighed in performance metrics can determine, therefore, whether human engagement is seen as a feature, a risk, or a cost.

### 4.4 Communication structures and role-filling

Our research found that some industry professionals draw on academic literature but others find themselves disconnected from cutting-edge research. At the same time, academics often lack an understanding of current industry activity and challenges. There are few outlets for researchers and engineers in industry positions to share technical challenges and evaluation techniques. Some commercial organizations contribute to the research community through sponsor soring competitions or participating in conferences. Sometimes, both industry professionals and academics are represented in working groups, such as those convened by government or international organizations (NIST, ISO, etc.) to discuss industry-wide standards. Industry can communicate research needs through design challenges, such as the Amazon Picking Challenge, which offer companies ways to engage researchers in cutting edge research problems and share some of the resources and data that the company has at its disposal. However, neither conferences, at which companies may only give high-level overviews of their work, nor working groups, which have limited participation, nor design challenges, which often largely benefit the sponsoring company, provide robust avenues of industry/academia exchange. Better and more systematic routes for exchange could inform industry professionals about academic innovations, direct academics to overlooked types of research problems, and offer the public greater information about the state of the field of product development, especially as robots have a greater presence in the human world.

Finally, our research highlights industry challenges and areas of preoccupation that are generally not the focus of academic labs. Yet, industry professionals understood that robotics is a field characterized by an ecosystem of actors. Building robots, particularly robots that will engage with the human world, is a complex challenge that requires a large cast of academic researchers and industry professionals with shared research goals, but different roles to play.

## 5 Limitations

As with all qualitative research, this study reflects the perspectives of a limited sample and the interpretations of its researchers. In addition to the limited sample size, we recognize that the absence of female representation among our participants is a major limitation to this work. This group could potentially have perspectives that are not captured in the data that was collected. While our team sought to mitigate bias through a cross-disciplinary approach and systematic analysis, our findings should be understood as exploratory. The 16 participants represented a range of professional experiences in mobile robotics, but a larger and more diverse sample could provide additional nuance. Nevertheless, the breadth of views captured here highlights recurring themes and core concerns across the industry, offering an initial typology to inform future HRI research and practice.

## 6 Conclusion

This study advances HRI by showing how engineers in commercial robotics conceptualize person detection and balance social interaction with safety and technical performance. Our interviews revealed approaches ranging from treating humans as generic obstacles to developing advanced classification models that differentiate among types of people. These strategies reflect not only technical capabilities but also how organizations prioritize between task completion, safety, innovation, and sociality. Prioritizing sociality can facilitate robot integration into human spaces, while extreme sociality, or prioritizing zero-risk HRI, may affect task completion, restrict robot deployment to controlled environments, or slow innovation. Industry professionals understood these trade-offs and articulated nuanced positive and negative effects. While our sample is necessarily limited, the analysis maps key tensions in industry practice: when human identification is treated as critical for safety versus when it is subsumed under performance optimization, and how contextual cues substitute for “acting socially” in constrained environments. By foregrounding these perspectives, we extend prior HRI research that has emphasized prototypes and controlled trials, offering instead a view of the practical considerations shaping commercial deployment.

For HRI researchers, these findings map the boundaries of current industry practice and highlight opportunities to refine evaluation metrics, address data asymmetries between academia and industry, and design robots that are both safe and socially cognizant. For practitioners, the typology we propose clarifies how person detection choices intersect with commercial readiness and deployment constraints. By situating industry perspectives within the broader HRI agenda, this work underscores the importance of bridging controlled experimental research with real-world design challenges. Future research should deepen this dialogue through larger-scale comparative studies and collaborative frameworks that integrate academic and industrial approaches.

## Data Availability

Due to confidentiality agreements, interview transcripts are not publicly available but anonymized excerpts may be provided upon reasonable request.
